# Supplementary material for: Machine learning for real-time aggregated prediction of hospital admission for emergency patients
Source: NPJ Digit Med. 2022 Jul 26;5:104. doi: 10.1038/s41746-022-00649-y (PMC9321296; doi:10.1038/s41746-022-00649-y)
Supplement: Supplementary file 2 — Reporting Summary [file 41746_2022_649_MOESM2_ESM.pdf]

nature portfolio

Corresponding author(s): Zella King

Last updated by author(s): Jun 30, 2022

Reporting Summary

Nature Portfolio wishes to improve the reproducibility of the work that we publish. This form provides structure for consistency and transparency in reporting. For further information on Nature Portfolio policies, see our Editorial Policies and the Editorial Policy Checklist.

Statistics

For all statistical analyses, confirm that the following items are present in the figure legend, table legend, main text, or Methods section.

n/a	Confirmed	
☐	☒	The exact sample size (n) for each experimental group/condition, given as a discrete number and unit of measurement
☒	☐	A statement on whether measurements were taken from distinct samples or whether the same sample was measured repeatedly
☒	☐	The statistical test(s) used AND whether they are one- or two-sided<br>*Only common tests should be described solely by name; describe more complex techniques in the Methods section.*
☒	☐	A description of all covariates tested
☒	☐	A description of any assumptions or corrections, such as tests of normality and adjustment for multiple comparisons
☒	☐	A full description of the statistical parameters including central tendency (e.g. means) or other basic estimates (e.g. regression coefficient) AND variation (e.g. standard deviation) or associated estimates of uncertainty (e.g. confidence intervals)
☒	☐	For null hypothesis testing, the test statistic (e.g. F, t, r) with confidence intervals, effect sizes, degrees of freedom and P value noted<br>*Give P values as exact values whenever suitable.*
☒	☐	For Bayesian analysis, information on the choice of priors and Markov chain Monte Carlo settings
☒	☐	For hierarchical and complex designs, identification of the appropriate level for tests and full reporting of outcomes
☒	☐	Estimates of effect sizes (e.g. Cohen's d, Pearson's r), indicating how they were calculated

Our web collection on statistics for biologists contains articles on many of the points above.

Software and code

Policy information about availability of computer code

Data collection	The research used an existing database created for other purposes (not specifically for this study). That database was created using custom computer programs which extracted data from various data warehouses routinely populated by the hospital's electronic health record system and from HL7 messages generated from hospital's electronic health record system
Data analysis	We are not able to provide the full code base as the hospital has asked us not to make SQL statements or other database-related information public for information security reasons. The remaining R code used for analysis and creation of the real-time application is available at https://github.com/zmek/real-time-admissions.

For manuscripts utilizing custom algorithms or software that are central to the research but not yet described in published literature, software must be made available to editors and reviewers. We strongly encourage code deposition in a community repository (e.g. GitHub). See the Nature Portfolio guidelines for submitting code & software for further information.

Data

Policy information about availability of data

All manuscripts must include a data availability statement. This statement should provide the following information, where applicable:

- Accession codes, unique identifiers, or web links for publicly available datasets
- A description of any restrictions on data availability
- For clinical datasets or third party data, please ensure that the statement adheres to our policy

The datasets analysed in this research are not publicly available. As a service evaluation, with the intended end point of deploying models for operational use, we were required to work with personally identifiable data throughout. Researchers wishing to validate or replicate this work using the same datasets would need to be approved for research collaborations with University College London Hospitals NHS Foundation Trust, and to secure appropriate permissions from the UCLH/UCL Joint Research Office. Researchers who meet these requirements can contact the corresponding author for further information about access to the datasets.

Human research participants

Policy information about studies involving human research participants and Sex and Gender in Research.

Reporting on sex and gender	The main study included a sample of 109,465 visits by adult patients to the emergency department of the hospital. The data on sex used in this study was that recorded in the patient record. 57,320 were recorded as female, 52,094 were male, and 51 had sex unspecified. A subsidiary study included 104,504 adult visits to the same department. In that study 53,678 were recorded as female, 50,784 as male and 58 unspecified.
Population characteristics	In the main study, mean (standard deviation) age was 43.1 (19.2). In the subsidiary study, mean (standard deviation) age was 44.8 (18.8). 25,259 further visits involving patients under 18 on the day of arrival, or where age was missing on the patient record were not included.
Recruitment	No recruitment was undertaken. All adult patients recorded in the database, which was created for a different purpose, were included.
Ethics oversight	The Joint Research Office of the university and the hospital (not named to respect the blind review process) deemed that the study was exempt from ethics review as there was no change to treatment or services or any study randomization of patients into different treatment groups

Note that full information on the approval of the study protocol must also be provided in the manuscript.

Field-specific reporting

Please select the one below that is the best fit for your research. If you are not sure, read the appropriate sections before making your selection.

☒ Life sciences ☐ Behavioural & social sciences ☐ Ecological, evolutionary & environmental sciences

For a reference copy of the document with all sections, see nature.com/documents/nr-reporting-summary-flat.pdf

Life sciences study design

All studies must disclose on these points even when the disclosure is negative.

Sample size	The main study included a sample of 109,465 visits by adult patients to the emergency department of the hospital. A subsidiary study included 104,504 adult visits to the same department.
Data exclusions	Children (aged less than 18 years) were excluded from the study because pediatric services were diverted away from the hospital during part of the study period due to the pandemic.
Replication	This paper describes the development of an analytical approach and an application in one hospital. The analytical approach was repeated on alternative datasets (derived from different periods of time) from the same hospital, as described in the Supplementary Note 6. The study is not directly replicable in other hospitals as database architectures will differ from one hospital to the next.
Randomization	Randomization was not appropriate as this was not an experimental study. The analysis was undertaken on all visits during the study period.
Blinding	Blinding was not relevant to this study as there were no experimental groups or conditions.

Reporting for specific materials, systems and methods

We require information from authors about some types of materials, experimental systems and methods used in many studies. Here, indicate whether each material, system or method listed is relevant to your study. If you are not sure if a list item applies to your research, read the appropriate section before selecting a response.

Materials & experimental systems

n/a	Involved in the study
☒	☐ Antibodies
☒	☐ Eukaryotic cell lines
☒	☐ Palaeontology and archaeology
☒	☐ Animals and other organisms
☒	☐ Clinical data
☒	☐ Dual use research of concern

Methods

n/a	Involved in the study
☒	☐ ChIP-seq
☒	☐ Flow cytometry
☒	☐ MRI-based neuroimaging
